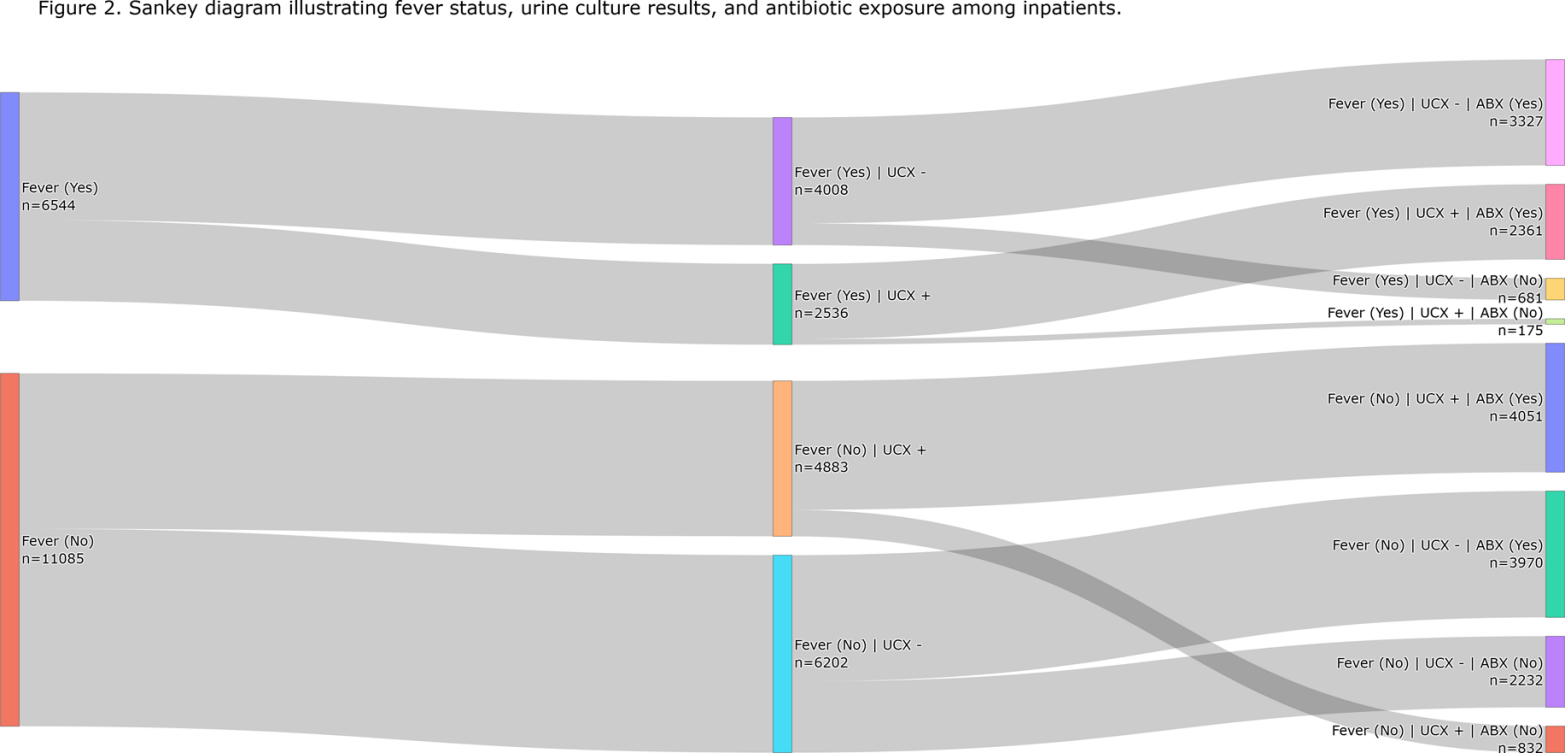# 182 Laboring Over Allergies: Assessing Penicillin Allergies and De-Labeling Potential in Obstetrics Population

**DOI:** 10.1017/ash.2026.10438

**Published:** 2026-06-23

**Authors:** Guillermo Rodriguez Nava, Rohana Bruker, Angela Montgomery, Marlee Barton, Jacob McAlinn, Timothy Jenkins, Heather Young

**Affiliations:** 1 Denver Health and Hospital Authority; 2 Denver Health Hospital Authority; 3 Denver Health; 4 Denver Health Medical Center

## Abstract

**Introduction:** Clinicians often order a variety of tests for hospitalized patients, including urinalysis with urine cultures. However, fever has limited diagnostic value for urinary tract infection (UTI); pooled estimates indicate that fever has likelihood ratios near 1, offering minimal discriminatory value for ruling UTI in or out. We evaluated the diagnostic yield of inpatient urine cultures by fever status in an acute care safety-net hospital in Denver, Colorado. **Methods:** We analyzed inpatient urine culture events from April 2016 through December 2025 using an automated electronic medical record report. Fever was captured within the National Healthcare Safety Network (NHSN) infection window period (+/- 3 calendar days). Culture positivity was defined per NHSN UTI criteria as ?10 colony forming units of one or two organisms; cultures with no growth or reported as yeast/Candida spp. or mixed flora were classified as negative. Antibiotic exposure was extracted from medications given up to 3 days after culture collection, and urinary infection-related diagnoses from problem/diagnosis entries up to 7 days after culture collection. To scale adjudication, we used deterministic text classification of medication and diagnosis fields. Medication strings were normalized and parsed with regular expressions to extract generic drug names (token preceding dose/formulation), then matched to a curated antibiotic dictionary. Urinary diagnoses were identified by regular-expression matching to UTI-related terms (e.g., UTI/urinary tract infection, cystitis, pyelonephritis, urosepsis). **Results:** Across 17,629 inpatient urine cultures, fever within the NHSN infection window period was documented in 37.1% of episodes and UTI diagnoses were documented in 29.6%. Overall, 7,983 cultures (42.1%) were positive for a bacterial pathogen, and antibiotics were prescribed in 77.8% of episodes. Culture positivity was similar to, or higher than, that among afebrile vs febrile episodes (44.1% vs 38.8%) and was higher with documented UTI diagnoses (58.5% vs 35.2% without). Culture positivity varied by strata: UTI diagnoses were more common among culture-positive episodes (60.9% with fever; 57.1% without fever), while febrile episodes without UTI diagnoses had the lowest positivity (29.4%) (Figure 1). Antibiotic exposure remained common across the fever–culture result combinations, including 22.5% of all episodes occurring in afebrile, culture-negative patients who nevertheless received antibiotics and 18.9% in febrile, culture-negative patients who received antibiotics (Figure 2). **Conclusion:** Fever was not reliably associated with urine culture positivity, whereas UTI diagnosis (clinical suspicion) was. Antibiotic use remained common after negative urine cultures, supporting opportunities to refine inpatient urine culture ordering and antimicrobial stewardship.

**Figure:**
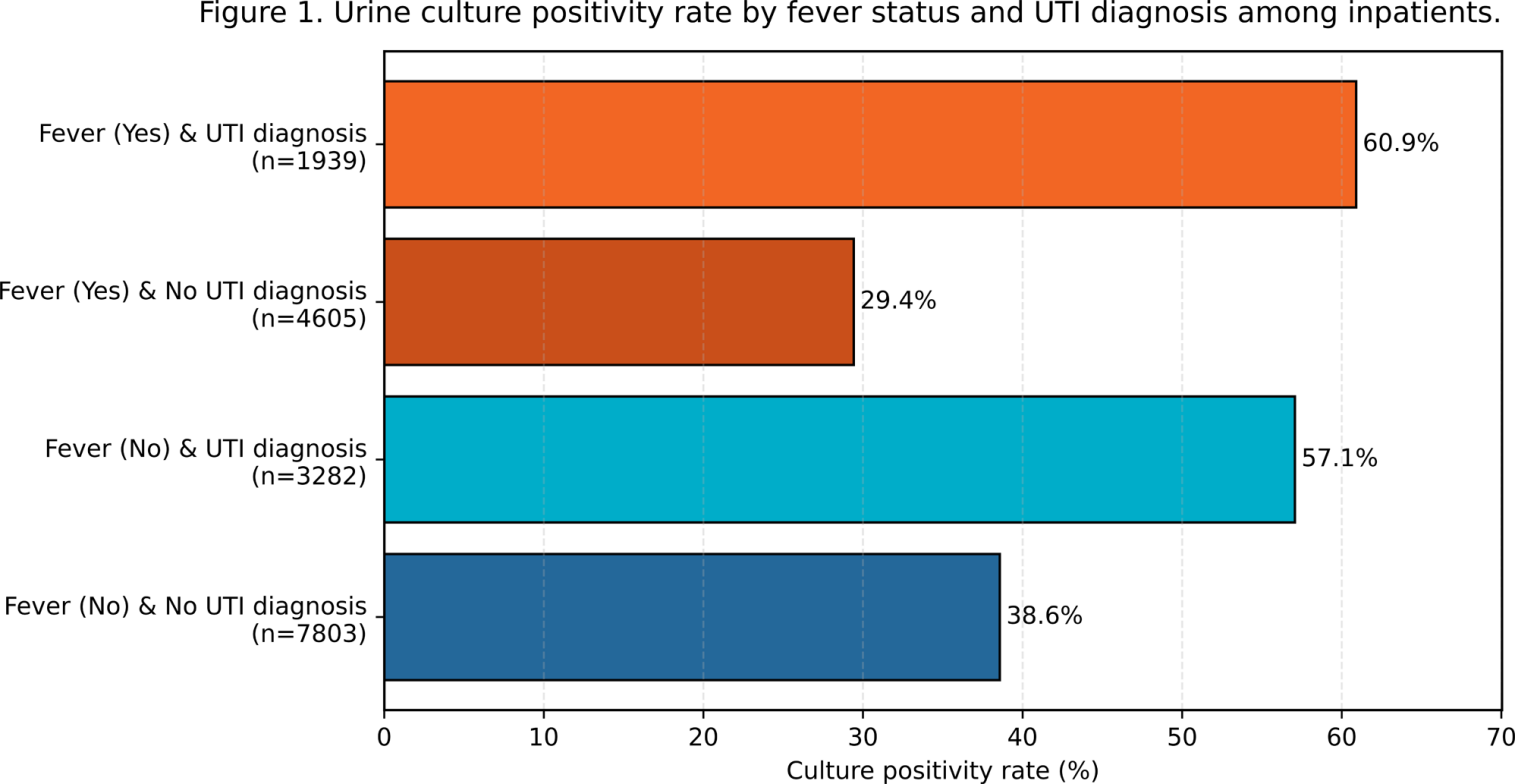

**Figure:**